# Sociodemographic Trends in Planetary Health Diets among Nutrition Students in Türkiye: Bridging Classroom to Kitchen

**DOI:** 10.3390/nu16091277

**Published:** 2024-04-25

**Authors:** Semra Navruz-Varlı, Hande Mortaş, Menşure Nur Çelik

**Affiliations:** 1 Department of Nutrition and Dietetics, Faculty of Health Sciences, Gazi University, Ankara 06490, Türkiye; semranavruz@gazi.edu.tr; 2 Department of Nutrition and Dietetics, Faculty of Health Sciences, Ondokuz Mayıs University, Samsun 55500, Türkiye; mensurenur.celik@omu.edu.tr

**Keywords:** planetary health diet, sustainable nutrition, diet quality, university students

## Abstract

This study aimed to investigate the effects of sociodemographic parameters on healthy and sustainable nutrition in nutrition students. This cross-sectional study was conducted with 601 students. Researchers administered questionnaire forms to gather sociodemographic information such as age, gender, geographical region, residence area, accommodation, BMI, and income level. Participants’ 24 h dietary records were used to evaluate Healthy Eating Index-2020 (HEI-2020) and Planetary Health Diet Index (PHDI). The mean PHDI scores of the Marmara (53.4 ± 14.9), Aegean (58.2 ± 18.3), Mediterranean (55.3 ± 15.5), and Black Sea (55.5 ± 15.7) regions, which are the coastal regions of Türkiye, were significantly higher than for the Central Anatolia region (46.7 ± 15.1). The PHDI and HEI-2020 score means of students living in metropolitan cities and rural areas were significantly higher than those living in urban areas (*p* < 0.05). Being in the 20–25 years age group increased the probability of being in a lower PHDI group (AOR 1.82; 95% CI 1.07:3.12; *p* = 0.028). While a similar result was found in the 20–25 years age group for HEI-2020, income level and gender did not have a statistically significant effect on these scores. Since students’ ages, geographical regions, and residence areas affect PHDI and HEI-2020, it is considered important to take these sociodemographic variables into consideration in guidelines and studies.

## 1. Introduction

It is known that the overweight and obesity situation seen in young adulthood is likely to continue or reach more serious levels in later adulthood [1]. During the university years, which represent a special and important part of young adulthood, social eating habits are shaped, which can significantly affect nutritional status and health in later periods of life. In this regard, it is important to evaluate the diet quality of university students, who are at a stage where the first steps are taken to a new life independent of the family and where basic responsibilities for life such as housing, subsistence, and nutrition are taken individually [2,3]. So, determining the dietary quality of university students, who constitute a significant portion of the young adult group, is important in preventing/controlling health problems that may arise related to nutrition. It is especially important because of their possible role as food preparers in their own future nuclear families and their potential to be role models for the children they will raise [4]. Among university students in Türkiye, the group of students that receive the most intensive nutrition-related education (4 years, theoretical and practical) are the students of departments of nutrition and dietetics [5]. It will be useful in making predictions about the diet quality and sustainable nutrition behavior trends of young people who continue their education in fields other than nutrition. This view is supported by the fact that studies have shown that university students who receive education in the field of health and nutrition are more likely to exhibit positive attitudes and behaviors related to nutrition than those who receive education in science and social fields [6]. In addition, there is a recent study showing that young German medical students who receive long-term nutrition education have better diet quality and more sustainable eating behaviors than the general population [7]. Therefore, determining the nutritional quality and sustainable nutrition behaviors of nutrition students, who were selected as a special subgroup among young adults in our study, is thought to be valuable in terms of evaluating the behavior of this special group, which will raise awareness in this field for the rest of the population.

Effectively implementing sustainable food policies entails addressing various sustainability issues and accommodating a diverse range of food systems, stakeholders, and consumer dietary behaviors [8,9]. Prioritizing the collection of data across these dimensions including sociodemographical issues and ensuring their seamless integration into models are crucial for advancing the sustainable diet agenda. Drawing on country-specific research findings regarding diet quality and sustainability can enhance the accuracy of assessments on the environmental impacts of consumer dietary behaviors in studies focused on sustainability [8,10]. In research conducted for this purpose [10,11,12,13], one of the most frequently used tools to evaluate the sustainability dimension of the diet is the Planetary Health Diet Index (PHDI), which is obtained by converting the sustainability and healthy nutrition principles in the EAT-Lancet reference diet into a numerical index [12]. The Planetary Health Diet promotes a high intake of plant-based foods and a low consumption of animal-based foods, following recommended portions for a 2500-kilocalorie daily diet [14]. 

Considering the statements of the Food and Agriculture Organization [15] that food production must increase in order to meet the needs of the increasing global population [16], it becomes inevitable that updated nutritional guidelines should be created by prioritizing the principle of sustainability. To justify the inclusion of sustainable dietary patterns in these guidelines, the need to compare the PHDI with health-oriented dietary recommendations currently in use has been reported [17]. One of the most commonly used indexes to compare the compliance of individuals’ diets with healthy eating recommendations is the Healthy Eating Index (HEI), which was developed to compare compliance with the Dietary Guidelines for Americans [18]. HEI-2020, the latest version of the updated index according to the guide, scores according to threshold values defined according to healthy nutrition recommendations but does not discourage the consumption of animal-sourced foods [17,18]. Additionally, for the HEI, nutritional differences have been demonstrated by gender, income, education, and race/ethnicity [19,20,21]. However, it is emphasized that the evidence for inequalities in PHDI is insufficient [10,17]. 

Increasing PHDI scores have been associated with improved diet quality and reductions in obesity and waist circumference [11]. In addition, levels of awareness about diet quality and planetary health tend to be higher in women than in men, in older age groups than young age groups, and in those with higher socioeconomic status than in those with lower socioeconomic status. The region of residence may have a positive or negative effect on diet quality and PHDI scores by restricting access to food or depending on income level [7,17,22].

When evaluating the reflections of differences such as gender, income, education, and ethnicity on the PHDI, there is a need to (1) present country-specific research results and, in doing so, to (2) present the results from the perspective of diet quality in order to add sustainability to existing nutritional recommendations. Therefore, this study aimed to investigate the effects of sociodemographic parameters on healthy and sustainable nutrition in nutrition students who have academic knowledge about sustainable and healthy nutrition approaches. In the study conducted for this purpose, it was hypothesized that sociodemographic variables including gender, age, income level, geographical region, BMI, and accommodation location affect healthy and sustainable nutrition.

## 2. Materials and Methods

### 2.1. Participants and Study Design

This cross-sectional study involved 601 adults, comprising 582 females and 19 males, who were enrolled as students in nutrition and dietetics programs at universities in Ankara, the capital city of Türkiye, from September 2023 to December 2023. Non-probabilistic convenience sampling was carried out. This study would require a total of 550 participants with 95% power at the 5% type I error (α) level. Participants were included if they volunteered and were university students studying in the nutrition and dietetics programs, did not follow a specific diet or eating regimen, and did not have any chronic diseases. Exclusions were made for individuals with a daily energy intake below 600 or above 3500 kilocalories according to 24 h dietary records, as well as for pregnant or lactating individuals. Additionally, if any data were missing in the data collection tool questionnaire, that individual was not included in the study.

Ethical approval was obtained from the Ethical Committee of Gazi University (Approval date: 14 July 2023, No: 2023-854). In addition, written informed consent was obtained from the participants in the study. The research was carried out following the Declaration of Helsinki. 

### 2.2. Data Collection Tools

In the study, researchers conducted face-to-face interviews with participants, administering questionnaire forms to gather sociodemographic information such as age, gender, regions where they spent their life until they went to university, residence area, accommodation, and income level. Additionally, participants were asked to complete 24 h dietary record forms for three consecutive days. Height and body weight measurements were self-reported. Body mass index (BMI) was defined as a body weight in kilograms divided by the square of the height in meters (kg/m^2^) [23]. 

Nutritional assessment was conducted based on the data collected from the dietary records. The nutritional content of the dishes consumed was calculated using the Standard Food Recipes book [24]. Subsequently, the data were analyzed using the BeBiS program (version 7.2) to assess total energy and nutrient intake. Participants’ nutritional intake was calculated by averaging the data obtained from 24 h dietary record forms for three consecutive days. 

The Planetary Health Diet Index (PHDI), devised by Cacau et al. (2021), is based on the dietary guidelines outlined by the EAT-Lancet Commission [12]. This index assesses adherence to these guidelines through a scoring system ranging from 10 to 5 points for each of the 16 diet components, with a total possible score of 0 to 150. Participants’ dietary records were used to evaluate these components. Based on their total PHDI score, participants are categorized into tertiles. 

The Healthy Eating Index (HEI) was established by the United States Department of Agriculture (USDA) in 1995, aligning with the American Dietary Guidelines [18]. Subsequent updates occurred in 2005, 2010, and 2015, maintaining consistency in components and standards between HEI-2015 and HEI-2020. Despite the name change to emphasize its association with the latest 2020–2025 Dietary Guidelines for Americans, HEI-2020 retains identical criteria and scoring standards to HEI-2015. The HEI-2020 utilized in this study comprises thirteen components, with nine recommended for consumption and four for limited intake. Components recommended for consumption include total fruit, whole fruit, total vegetables, green leafy vegetables and legumes, whole grains, dairy products, protein foods, seafood and plant-based proteins, and fatty acids. Moderation is advised for processed grains, sodium, added sugar, and saturated fats. Higher total scores indicate better nutritional quality across all components [18]. Based on their total HEI-2020 score, participants are categorized into tertiles. 

### 2.3. Statistical Analysis

Continuous variables were expressed as arithmetic mean with standard deviation and categorical variables as percentages. The HEI-2020 total score and PHDI total score were compared according to age groups (<20 years; 20–25 years; >25 years), BMI groups (underweight; normal weight; overweight; obese), gender (female and male), regions of Türkiye (Marmara, Aegean, Mediterranean, Black Sea, Central Anatolia, Eastern Anatolia, Southeast Anatolia), residence areas (metropolis, urban, rural), income level (low, those whose expenses are more than their income; adequate, those whose expenses are equal to their income; high, those whose income exceeds their expenses), and accommodation status (dormitory and home) of the participants. Participants’ dietary energy, macronutrient, and micronutrient intakes were shown according to the income levels. 

The *t*-test (in Table 1 for gender and accommodation groups) and one-way ANOVA (in Table 1 for age groups, geographical regions, residence areas, income level groups, and BMI categories; in Table 2 for income level groups) were employed for comparisons in independent groups. Post hoc analysis involved the application of Bonferroni correction for handling multiple pairwise comparisons. Moreover, a series of multivariate ANOVAs (MANOVAs) were conducted with four sociodemographic variables which were found to be significantly different among groups according to Table 1 as independent variables and with the total scores of PHDI and HEI-2020 as dependent variables. The possible effect of age groups (<20 years; 20–25 years; >25 years), geographical regions (Marmara, Aegean, Mediterranean, Black Sea, Central Anatolia, Eastern Anatolia, Southeast Anatolia), residence areas (metropolis, urban, rural), and income level (low; adequate; high) on total scores of the PHDI and the HEI-2020 was analyzed using a multivariate analysis of variance (MANOVA) and the interaction among the factors using the Bonferroni statistic. Furthermore, the effect size was calculated in terms of eta squared (η^2^). The results of the MANOVAs are presented in Supplementary Material Tables S1 and S2. The four independent variables included age groups, geographical regions, residence areas, and income level. Of the four sociodemographical variables considered, one to four were entered into the MANOVA at a time, with combinations of sociodemographical variables selected such that cell sizes equaled or exceeded 30 (i.e., sufficient cell size to ensure normalcy of distribution of individual differences). When significant interactions were found, the file was split by both variables and MANOVAs were conducted with the other variable and only the significant findings reported. Parametric statistics were used to confirm the effects obtained via the MANOVAs when Levene’s test for homogeneity of variance was significant at the *p* > 0.05 level. 

Furthermore, a multivariable logistic regression model (in Table 3 and Table 4) was used to identify independent predictors, including dietary energy intake, genders, age groups, income levels, and residence area, of low planetary diet quality defined as T_1_. The odds of being in the low planetary diet quality group due to “being female”; “being in <20 years age group”; “having a high income level”; and “living in a metropolis” were examined; and for continuous variables specific intervals were used. The model fit was assessed using appropriate residual and goodness-of-fit statistics. A 5% type I error level was used to infer statistical significance. The IBM Statistical Package for the Social Sciences (SPSS) 28.0.1.0 program was used for statistical analysis, and significance was evaluated at *p* < 0.05.

## 3. Results

PHDI and HEI-2020 total scores according to individuals’ sociodemographic characteristics are shown in Table 1. The mean PHDI and HEI-2020 total scores of the participants were determined as 50.9 ± 15.6 and 47.9 ± 11.7, respectively. There was no statistical difference between the total PHDI and HEI-2020 scores of the participants according to their gender (*p* = 0.748 and *p* = 0.979, respectively). According to age groups, PHDI total score averages of students younger than 20 (54.2 ± 15.4) were significantly higher than for other age groups (48.8 ± 15.3 and 53.3 ± 16.1, respectively, in 20–25 years and >25 years groups; *p* < 0.001). In total HEI-2020 scores, the mean of the >25 years group (51.4 ± 11.8) was found to be statistically higher than that of the 20–25 years group (46.5 ± 12.0; *p* = 0.001) but was not different from the <20 years group (49.9 ± 10.7). 

Statistically significant differences were found between Türkiye’s total PHDI and HEI-2020 score means according to geographical region. The mean PHDI total scores of the Marmara (53.4 ± 14.9), Aegean (58.2 ± 18.3), Mediterranean (55.3 ± 15.5), and Black Sea (55.5 ± 15.7) regions, which are the coastal regions of Türkiye, were significantly higher than for the Central Anatolia region (46.7 ± 15.1; *p* < 0.001), without any difference among coastal regions. According to geographical region, HEI-2020 total mean scores were shown to be highest in the Southeast Anatolia region (52.8 ± 10.5) and lowest in the Central Anatolia region (45.4 ± 11.7; *p* = 0.001).

The total PHDI and HEI-2020 score means of students living in metropolitan cities and rural areas were significantly higher than those living in urban areas (*p* < 0.001). The mean PHDI scores of individuals in the “adequate” income group (48.4 ± 15.2), whose income was defined as equal to their expenses, were significantly lower than those of individuals in the “high” (54.5 ± 15.6) and “low” (53.8 ± 15.6) income groups (*p* < 0.001). The mean HEI-2020 scores in the “adequate” group (46.9 ± 12.1) were significantly lower than those in individuals in the “low” income group (49.5 ± 10.6; *p* = 0.038) but do not differ from individuals in the “high” income group (48.9 ± 12.1; *p* > 0.05). 

There were no statistically significant differences in the students’ PHDI and HEI-2020 total mean scores according to their dormitory or home accommodation status (*p* = 0.262 and *p* = 0.918, respectively) and BMI categories (*p* = 0.059 and *p* = 0.876, respectively).

The results of the MANOVAs are presented in Supplementary Material Tables S1 and S2. The four independent variables included age groups, geographical regions, residence areas, and income level. In almost all cases, the results of MANOVAs were confirmed the findings of the ANOVAs. In those cases, the results of the MANOVAs only are reported in Table S2. In cases where significant findings were not found on the tests they were not reported. There are no significant multivariate effects for the variables’ combinations in Table S1. Significant multivariate effects are found for the age groups (F (1, 2) = 0.684, *p* < 0.001, η^2^ = 0.003, CI, 51.106, 59.005), regions (F (1, 5); (2, 5); (3, 5); (4, 5) = 1.265, *p* = 0.038, η^2^ = 0.015, CI, 48.672, 57.012 for 1; 53.099, 66.549 for 2; 50.798, 61.886 for 3; 52.202, 65.242 for 4; 44.768, 53.199 for 5), residence areas (F (1, 2); (2, 3) = 0.696, *p* < 0.001, η^2^ = 0.003, CI, 47.811, 55.824 for 1; 50.402, 58.325 for 2; 52.137, 58.959 for 3), and income levels (F (1, 2); (2, 3) = 1.701, *p* = 0.006, η^2^ = 0.007, CI, 52.721, 61.594 for 1; 49.480, 55.431 for 2; 49.066, 57.389 for 3) in total PHDI scores in Table S2. The large number of samples resulted in smaller effect sizes, i.e., η^2^ values. The findings confirmed from Table 1 are shown in Table S2. 

Table 2 is presented to reveal whether there are significant differences in energy and nutrient intakes according to participants’ income levels. Participants’ energy and nutrient intakes according to their income level are shown in Table 2. Mean intakes of energy, protein, cholesterol, saturated fatty acids, fiber, thiamine, niacin, folate, vitamin C, iron, phosphorus, calcium, potassium, zinc, magnesium, and copper were found to be significantly higher in the “low” income group than those in the “adequate” and “high” income groups (*p* < 0.05). There was no statistical difference between income level groups in terms of individuals’ mean intake of carbohydrates (*p* = 0.057), fat (*p* = 0.395), vitamin A (*p* = 0.872), and vitamin B_12_ (*p* = 0.338).

In Table 3, when individuals are divided into tertiles according to their PHDI scores (low (T_1_)–medium (T_2_)–high (T_3_)), the risks of being in the lowest tertile category are shown with adjustment (adjusted odds ratio—AOR) and without adjustment (crude odds ratio—COR) according to various sociodemographic characteristics. For categorical variables with an OR higher than one, the odds of being in the T_1_ PHDI group were higher than in the reference group. For continuous variables, these odds became higher per specified interval. The probability of being in the T_1_ PHDI group increased with increasing dietary energy intake (kcal) (AOR 1.01; 95% CI 1.00:1.01; *p* = 0.01). Being in the 20–25 years age group increased the probability of being in the T_1_ PHDI group compared to the reference of the <20 years age group (AOR 1.82; 95% CI 1.07:3.12; *p* = 0.028).

In Table 4, when individuals are divided into tertiles according to their HEI-2020 scores (low (T_1_)–medium (T_2_)–high (T_3_)), the risks of being in the lowest tertile category are shown with adjustment (adjusted odds ratio—AOR) and without adjustment (crude odds ratio—COR) according to various sociodemographic characteristics. For categorical variables with an OR higher than one, the odds of being in the T_1_ HEI-2020 group were higher than in the reference group. For continuous variables, these odds became higher per specified interval. Being in the urban residence area group increased the probability of being in the T_1_ HEI-2020 group compared to the reference of the metropolis residence area group (AOR 1.83; 95% CI 1.15:2.91; *p* = 0.011).

**Figure 1:**
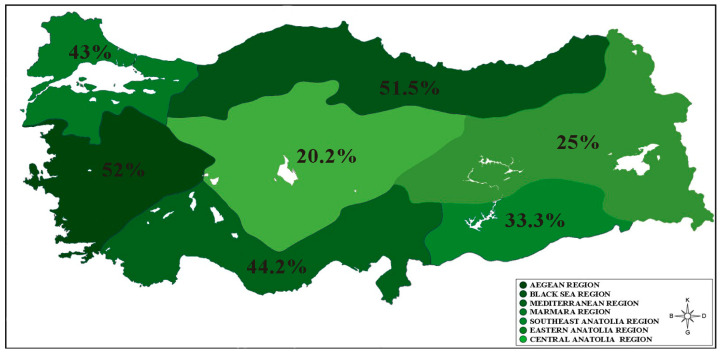
The percentage distribution of individuals in the PHDI T_3_ group according to Türkiye’s geographical regions.

The percentage distribution of individuals in the T_3_ group, which is the highest tertile according to individuals’ PHDI score tertiles, in the geographical regions of Türkiye is shown in Figure 1. Geographic regions indicated in darker green indicate higher percentages of participants in the higher PHDI tertile (T_3_) in these regions. So, dark green colors visualize higher percentages, and as the green tone becomes lighter, the percentage of individuals in the T_3_ tertile decreases. It was found that the individuals with the highest PHDI scores (T_3_) were in the Aegean (52%), Black Sea (51.5%), Mediterranean (44.2%), and Marmara (42%) regions of Türkiye, expressed in darker green. These regions were followed by Southeast Anatolia (33.3%), Eastern Anatolia (25%), and Central Anatolia (20.2%). 

**Figure 2:**
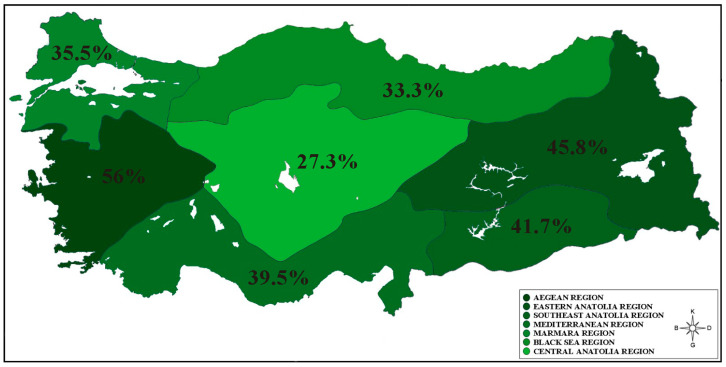
The percentage distribution of individuals in the HEI-2020 T_3_ group according to Türkiye’s geographical regions.

The percentage distribution of individuals in the T_3_ group, which is the highest tertile according to individuals’ HEI-2020 score tertiles, in the geographical regions of Türkiye is shown in Figure 2. Geographic regions indicated in darker green indicate higher percentages of participants in the higher HEI-2020 tertile (T_3_) in these regions. So, dark green colors visualize higher percentages, and as the green tone becomes lighter, the percentage of individuals in the T_3_ tertile decreases. It was found that the individuals with the highest HEI-2020 scores (T_3_) were in the Aegean (56%), Eastern Anatolia (45.8%), Southeast Anatolia (41.7%), and Mediterranean (39.5%) regions of Türkiye, expressed in darker green. These regions were followed by Marmara (35.5%), Black Sea (33.3%), and Central Anatolia (27.3%). 

**Figure 3:**
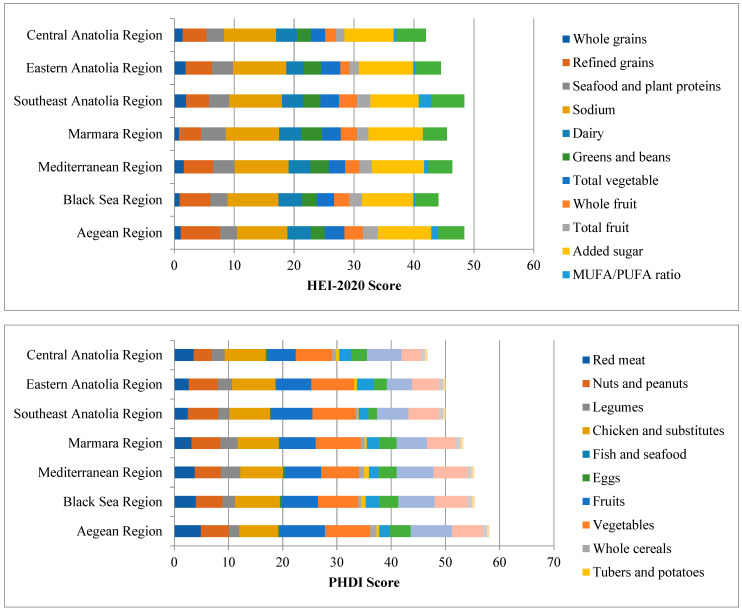
HEI-2020 and PHDI component scores according to Türkiye’s regions.

Participants’ HEI-2020 and PHDI component scores according to Türkiye’s regions are presented in Figure 3. Among the HEI-2020 components, the total vegetable score was highest in the Aegean, Mediterranean, and Black Sea regions, which are the coastal areas of Türkiye. Similarly, seafood and plant protein scores were highest in these regions, while these scores were low in the Central Anatolia and Eastern Anatolia regions. It was seen that the score in the dairy group was highest in the Southeast Anatolia region. 

Among the PHDI components, the lowest red meat score was found in the Southeast Anatolia and Eastern Anatolia regions. It was visualized that the fish and seafood scores were lowest in the Central Anatolia and Eastern Anatolia regions and, similarly, the vegetable and whole cereals scores were lowest in these regions. 

## 4. Discussion

This study endeavors to explore the impact of sociodemographic factors on the adoption of sustainable and healthy dietary practices among nutrition students well-versed in the principles of sustainable nutrition. To the best of our knowledge, this is the first study to examine PHDI, a nutritional index designed to account for both environmental and health concerns, in Turkish adults in addition to HEI-2020, a nutritional index designed to account only for health concerns, and to assess how well their diets adhered to PHDI. This study found low compliance with EAT-Lancet recommendations and low PHDI scores across all of our geographic regions. It has been shown that age, income level, geographical region, and place of residence are the components that affect the PHDI score. In comparison to other places, high PHDI scores were found in our coastal areas.

Although the PHDI has been tested in the United States population, it is designed for use in a variety of settings. Global diets are neither as healthy nor as sustainable as the EAT-Lancet Commission reference diet. However, it is stated that heterogeneity in how these diets differ from recommendations can be determined by PHDI. Application of the PHDI in diverse global settings could provide a unified framework for directly comparing the health and sustainability of nutrition across countries and monitoring progress over time [25].

PHDI is associated with higher overall nutritional quality and lower greenhouse gas emissions [12,26]. In a study conducted in the Turkish population, it was shown that the average PHDI score was 41.5 when evaluated according to PHDI quartile distributions [10]. In a study conducted in Brazil, PHDI scores were found to be similar (45.9 points) and society’s compliance with EAT-Lancet recommendations was evaluated as low [13]. In another study conducted by Cacau et al. in the Brazilian population, the average PHDI score was 60.4, which seems relatively high compared to other countries, but is far from meeting the recommendations [11]. In a recent study, mean PHDI was lower (*p* < 0.001) in men than in women (44.0 and 47.1, respectively) and tended to be lower in younger individuals [17]. On the contrary, in this study, there was no statistical difference between the total PHDI scores of the participants according to their gender, and the average PHDI total score of the participants under the age of 20 was significantly higher than that of the other age groups (Table 1). According to data obtained from all regions of our country, the diet of our population appears to be far from being in line with the evidence presented by EAT-Lancet. It can be said that the average PHDI scores obtained in this study are less than half of the maximum score of 150, and compliance with EAT-Lancet recommendations is low in our country (Table 1). Our results are parallel to previous studies and literature in our country in terms of PHDI score. The fact that PHDI average scores are high, especially in coastal regions (Marmara, Black Sea, Aegean, and Mediterranean) (Table 1, Figure 1 and Figure 2), may be related to the diversity of foods especially food groups such as fish and seafood, vegetables, fruits, dairy, and eggs, which are among the “adequacy” and “optimum” components in the PHDI score, consumed in these regions (Figure 3). In addition, in this study, when the low planetary health diet quality of the participants was compared with the average–high planetary health diet quality according to different variables, being between the ages of 20 and 25, living in a rural area, and increased energy intake were found to be important risk factors for a decrease in the PHDI score (Table 3).

For PHDI to be widely adopted, it must be acceptable to consumers. While there are various factors that influence consumer food choices, such as accessibility, availability, health concerns, and food preferences [27,28], the role of affordability is also considered a key factor that can influence PHDI score [29,30]. While health and sustainability are desirable outcomes of consumer choices, affordability often takes priority, especially for low-income consumers [30,31,32]. Therefore, it is necessary to understand the cost and affordability of a healthy and sustainable diet such as used in the PHDI for various socioeconomic groups. Two studies conducted in the United Kingdom determined the cost of a healthy, sustainable diet and compared this to the typical diet consumed in that country, finding that there was no increase in cost for following a healthy, sustainable diet [33,34]. On the contrary, in one study conducted in Australia, the typical Australian diet was found to be more costly than a healthy and sustainable diet [35], while in another, the result was found to be the opposite [29]. A PHDI-compliant diet has been shown to be affordable for high-income countries but unaffordable for low-income countries [36]. PHDI and HEI-2020 were found to be higher in those with “low” and “high” income levels and living in “rural” and “metropolitan” areas than those with “sufficient” income levels and living in “urban” areas (*p* < 0.05) (Table 1). When dietary intakes are examined (Table 2), significantly higher saturated fat, cholesterol, phosphorus, and iron intakes in the low-income group show that consumption of animal-based foods is high. However, their high fiber intake suggests that plant sources are included in the diet more than in individuals with other income levels, which positively affects PHDI scores. It is possible that these individuals are individuals living in rural areas. It is thought that this finding was obtained because individuals living in rural areas in our country frequently engage in animal husbandry and the consumption of animal products is common among these individuals. Our findings support our idea that those with high income levels live in metropolises. Although dietary energy intake is low, PHDI and HEI-2020 scores are thought to be high in those living in metropolises due to access to sufficient resources. Individuals with sufficient income levels seem to live in urban areas. Considering city life and daily work pace, it is thought that their dietary intake is not sufficient compared to rural areas, and therefore HEI-2020 and PHDI scores remain low. In addition, although individuals with sufficient and high income levels have similar energy, carbohydrate, fat, and micronutrient intakes, when looking at their diet patterns, it is possible to say that protein intake remains lower at sufficient income levels, and this affects the diet quality of individuals. Considering that the sufficient income group lives in urban areas and the high-income group lives in metropolises, factors such as limited access to resources in urban areas compared to metropolises and the lower income level of those living in urban areas than individuals in metropolitan areas may explain the lower HEI-2020 and PHDI scores in individuals with sufficient income compared to high income levels.

In a study evaluating the obesity consequences of compliance with PHDI, it was observed that individuals with higher compliance with PHDI had lower BMI and waist circumference values [11]. However, another study showed that overweight/obese individuals had higher PHDI scores [13]. One study determined that higher BMI was associated with lower PHDI, with a decrease in BMI associated with an increase in PHDI total scores [10]. Therefore, it is thought that more studies are needed on the effects of obesity on the sustainable environment and the effects of sustainable nutrition on the prevalence of obesity. In this study, no statistically significant difference was found in the PHDI and HEI-2020 total score averages according to BMI categories. Although not statistically significant in the group classified as normal BMI, lower PHDI scores are noteworthy. It is thought that statistical significance may not have been achieved because the majority of the participants in the study (76.7%) were in the normal BMI class (Table 1 and Table 2).

Country-specific studies are important due to different cultures, traditions, and access to food in each country, as well as different environmental factors in each country [8]. It is important that this healthy and sustainable diet model is addressed in a way that covers all regions in our country in the context of revealing local differences.

While some studies report that no relationship can be found between dietary energy, protein, and PHDI scores [10,12], the findings of some studies that dietary fiber positively affects nutritional scores are noteworthy [10,37]. Another study also showed that sustainable and healthy eating behaviors and high adherence to PHDI increased with dietary fiber intake [38]. Similarly, in this study, individuals in the “low” income group, whose dietary fiber and folate intake was significantly higher than for other income levels, were found to have higher PHDI scores (Table 1 and Table 2).

Low PHDI scores are due to several components. Consumption of moderation components in the United States exceeds targets for added sugars, added fats, saturated fats, trans fats, dairy products, and red and processed meats, reflecting the typical “Western” dietary pattern [25]. This is consistent with other findings regarding animal-derived food consumption in the United States [39]. In addition to overconsumption of moderation ingredients, underconsumption of various adequacy ingredients such as whole grains, fruits, vegetables, legumes, nuts, and seeds has also been reported. Adherence to fruit and vegetable recommendations has been low in the United States [25]. Similar to a cross-sectional study conducted in Brazil [12], adolescents who consumed excessive amounts of animal-derived foods scored lower on the PHDI [12,14]. The scores obtained from the HEI-2020 and PHDI components have an expected distribution in terms of geographical regions in our country. It is expected that seafood and vegetable consumption, and therefore the scores obtained from their consumption, will be high due to agricultural and fishing activities in coastal areas. While the scores obtained from the milk and meat groups were expected to be high in the Eastern and Southeastern Anatolia regions, as they are the regions where the main livestock activities of our country are carried out, the lowest red meat scores were found in the Southeastern Anatolia region. As expected, the scores obtained from milk and its products are highest in the Eastern Anatolia region. Although the Central Anatolia region is described as a “granary”, the score obtained from this food group remained low (Figure 3).

The following limitations should be taken into account when evaluating the research findings. It should be kept in mind that this cross-sectional study cannot clearly reveal the cause–effect relationship and can only help us make inferences by providing information about the current situation. The participants’ weight and height are based on self-report, which may be misleading. Additionally, supplement use was not taken into account in nutritional intake. However, the goal of EAT-Lancet is to provide a nutritionally adequate diet without the need for nutritional supplements. In this study, we evaluated the situation regarding nutritional intake. Additionally, one of the limitations of the study is that the participants were not distributed evenly in terms of gender. The fact that males are more reluctant than females to participate in research and that the nutrition department students in Türkiye are predominantly female has led to this result. From this perspective, the gender distribution in the study also reflected the distribution of nutrition students in Türkiye. However, the inclusion of only a specific group in the study and the imbalance in gender distribution limit the generalizability of the results to the whole population of Türkiye.

The strengths of the study are as follows: The fact that it evaluated the relationship between diet quality and planetary health across the country suggests that the research sample may reflect Türkiye in terms of sociodemographic variables. In addition, it is important that it is one of the first studies to evaluate individuals’ sustainable healthy eating behaviors and the compliance of their diets with PHDI on a regional basis and that it considers PHDI together with HEI-2020, another important dietary index. One of the strengths of the study is that the 24 h recording method, which is thought to be more accurate than the food consumption frequency questionnaire, was used to calculate PHDI. Additionally, although the fact that the study was conducted in the nutrition department as a specific group may seem like a limitation, we think that sustainable and healthy nutrition is low even in a population with basic knowledge about nutrition and that it is valuable to reveal sociodemographic variables.

## 5. Conclusions

This study seeks to understand how individuals’ background influences their choices about eating healthily and sustainably, focusing on nutrition students who are familiar with these concepts. In conclusion, to our knowledge, this study is among the first articles to analyze adherence to the EAT-Lancet universal healthy reference diet by geographical region in a nationally representative sample of adults in Türkiye. Revision of the diet in our country according to EAT-Lancet recommendations will increase nutritional adequacy in terms of some food groups and nutrients. This research shows that participants’ PHDI index scores were low. Therefore, adherence to the EAT-Lancet recommendation was determined to be low. No association of BMI with lower PHDI scores was found. However, it was concluded that age, income level, geographical region, and place of residence affect PHDI scores and HEI-2020 scores similarly. Policies are needed to transform food systems and create a more sustainable nutrition pattern. For this reason, in creating sustainable and healthy nutrition behaviors, the importance of developing nutrition guidelines by evaluating the environmental factors affecting them not only across the country but also in geographical and sociodemographic clusters within the same country should not be ignored by policy developers. 

It is important for future studies to be conducted on a larger sample that is homogeneously distributed in terms of sociodemographic variables that can represent the country in order to generalize the results. In addition, future studies will be able to reveal the causality between PHDI and sociodemographic variables through longitudinally planned studies. It is thought that this cross-sectional study will be a guide in this direction.

## Figures and Tables

**Table 1:** Healthy Eating Index-2020 and Planetary Health Diet Index total scores of the students according to the sociodemographic variables.

Variables	Proportion [*n* (%)]	Total PHDI Score			Total HEI-2020 Score		
		Mean ± SD	95% CI		Mean	95% CI	
Total	601 (100)	50.9 ± 15.6	49.7	52.2	47.9 ± 11.7	47.0	48.9
Gender							
Females	582 (96.8)	50.8 ± 15.5	49.6	52.2	47.9 ± 11.6	47.1	48.9
Males	19 (3.2)	52.1 ± 18.1	43.4	60.8	48.0 ± 12.6	41.9	54.1
		t = 0.321 *p* = 0.748			t = 0.026 *p* = 0.979		
Age group							
<20 years	215 (35.8)	54.2 ± 15.4 ^a^	52.1	56.2	49.9 ± 10.7 ^a,b^	48.4	51.3
20–25 years	354 (58.9)	48.8 ± 15.3 ^b^	47.2	50.4	46.5 ± 12.0 ^b^	45.3	47.8
>25 years	32 (5.3)	53.3 ± 16.1 ^a,b^	47.5	59.1	51.4 ± 11.8 ^a^	47.1	55.6
		F = 8.635 *p* < 0.001			F = 7.135 *p* = 0.001		
Regions							
Marmara	214 (35.6)	53.4 ± 14.9 ^a^	51.4	55.4	49.3 ± 10.4 ^a^	47.9	50.7
Aegean	25 (4.2)	58.2 ± 18.3 ^a^	50.7	65.7	52.1 ± 12.6 ^a,b^	46.9	57.3
Mediterranean	43 (7.2)	55.3 ± 15.5 ^a^	50.5	60.0	50.2 ± 13.5 ^a,b^	46.1	54.4
Black Sea	33 (5.5)	55.5 ± 15.7 ^a^	49.9	61.0	47.6 ± 13.6 ^a,b^	42.8	52.1
Central Anatolia	238 (39.5)	46.7 ± 15.1 ^b^	44.8	48.6	45.4 ± 11.7 ^b^	43.9	46.9
Eastern Anatolia	24 (4.0)	50.0 ±14.3 ^a,b^	43.9	56.1	48.9 ± 10.5 ^a,b^	44.1	52.9
Southeast Anatolia	24 (4.0)	50.2 ± 15.5 ^a,b^	43.7	56.7	52.8 ± 10.5 ^a^	47.8	57.9
		F = 6.096 *p* < 0.001			F = 3.947 *p* = 0.001		
Residence area							
Metropolis	265 (44.1)	52.5 ± 15.8 ^a^	50.6	54.5	49.7 ± 10.9 ^a^	48.4	51.0
Urban	215 (35.8)	47.3 ± 15.2 ^b^	45.2	49.3	44.7 ±11.3 ^b^	43.2	46.3
Rural	121 (20.1)	53.9 ± 14.4 ^a^	51.4	56.6	49.9 ± 12.5 ^a^	47.6	52.2
		F = 9.957 *p* < 0.001			F = 13.347 *p* < 0.001		
Income level							
Low	200 (33.3)	53.8 ± 15.6 ^a^	51.6	55.9	49.5 ± 10.6 ^a^	47.9	50.9
Adequate	328 (54.6)	48.4 ± 15.2 ^b^	46.8	50.0	46.9 ± 12.1 ^b^	45.6	48.2
High	73 (12.1)	54.5 ± 15.6 ^a^	50.9	58.1	48.9 ± 12.1 ^a,b^	46.0	51.7
		F = 9.920 *p* < 0.001			F = 3.285 *p* = 0.038		
Accommodation							
Dormitory	493 (82.0)	50.6 ± 15.6	49.2	51.9	47.9 ± 11.7	46.9	48.9
Home	108 (18.0)	52.5 ± 15.7	49.5	55.5	48.1 ± 11.7	45.8	50.3
		t = −1.122 *p* = 0.262			t =−0.103 *p* = 0.918		
BMI categories							
Underweight	52 (8.7)	56.1 ± 16.6	51.4	60.7	49.2 ± 12.8	45.6	52.7
Normal weight	461 (76.7)	50.2 ± 15.4	48.8	51.6	47.9 ± 11.6	46.9	48.9
Overweight	70 (11.6)	51.5 ± 16.0	47.6	55.3	47.5 ± 11.4	44.8	50.2
Obese	18 (3.0)	53.8 ± 12.5	47.6	60.1	47.9 ± 12.5	41.6	54.1
		F = 2.492 *p* = 0.059			F = 0.230 *p* = 0.876		

CI: Confidence interval; BMI: Body mass index; HEI: Healthy Eating Index; PHDI: Planetary Health Index; SD: Standard deviation. ^a,b^ represent the statistically significant differences among the column groups at *p* < 0.05.

**Table 2:** Dietary energy, macronutrient, and micronutrient intakes of the students according to income level.

Dietary Intake ( $\bar{x}$ ± SD)	Income Level			F	*p*
	Low	Adequate	High		
Energy (kcal)	1580.1 ± 589.7 ^a^	1343.9 ± 501.1 ^b^	1370.5 ± 591.7 ^b^	12.165	**<0.001**
Carbohydrates (% of energy)	43.8 ± 9.6	45.9 ± 11.6	43.3 ± 10.7	3.328	0.057
Proteins (% of energy)	15.6 ± 4.6 ^a^	14.5 ± 4.6 ^b^	16.0 ± 6.3 ^a^	5.023	**0.007**
Fats (% of energy)	40.7 ± 8.9	39.5 ± 10.8	40.8 ± 10.0	0.930	0.395
Cholesterol (mg)	273.7 ± 14.7 ^a^	215.2 ± 9.8 ^b^	221.9 ± 16.5 ^a,b^	6.508	**0.002**
Saturated fat (g)	24.2 ± 13.3 ^a^	19.2 ± 10.1 ^b^	20.9 ± 11.7 ^a,b^	11.791	**<0.001**
Fiber (g)	17.5 ± 8.4 ^a^	14.3 ± 7.5 ^b^	14.7 ± 8.7 ^b^	10.068	**<0.001**
Thiamine (mg)	0.8 ± 0.3 ^a^	0.7 ± 0.3 ^b^	0.7 ± 0.2 ^a,b^	11.407	**<0.001**
Riboflavin (mg)	1.1 ± 0.5 ^a^	0.9 ± 0.6 ^b^	1.0 ± 0.4 ^a,b^	4.098	**0.017**
Niacin (mg)	11.3 ± 6.9 ^a^	9.6 ± 7.4 ^b^	10.4 ± 6.3 ^a,b^	3.589	**0.028**
Folate (mcg)	269.9 ± 12.9 ^a^	203.7 ± 11.8 ^b^	229.3 ± 13.1 ^b^	17.519	**<0.001**
Vitamin A (mcg)	935.3 ± 57.1	829.8 ± 168.4	906.7 ± 96.1	0.137	0.872
Vitamin B_12_ (mcg)	3.7 ± 2.7	3.2 ± 5.2	3.1 ± 2.5	1.087	0.338
Vitamin C (mg)	101.6 ± 78.9 ^a^	70.8 ± 61.7 ^b^	83.8 ± 59.1 ^a,b^	12.960	**<0.001**
Iron (mg)	9.5 ± 5.3 ^a^	7.4 ± 3.8 ^b^	7.9 ± 4.3 ^a,b^	14.708	**<0.001**
Phosphorus (mg)	930.4 ± 353.9 ^a^	785.4 ± 353.3 ^b^	802.7 ± 307.6 ^b^	11.140	**<0.001**
Calcium (mg)	552.7 ± 264.9 ^a^	482.1 ± 305.6 ^b^	503.7 ± 287.8 ^a,b^	3.724	**0.025**
Potassium (mg)	2180.6 ± 902.6 ^a^	1696.4± 844.8 ^b^	1855.688.8 ^b^	20	**<0.001**
Zinc (mg)	8.3 ± 3.4 ^a^	6.8 ± 3.4 ^b^	6.7 ± 3.2 ^b^	733	**<0.001**
Magnesium (mg)	239.6 ± 103.9 ^a^	185.7 ± 87.1 ^b^	203.7 ± 89.2 ^b^	20.291	**<0.001**
Copper (mg)	1.2 ± 0.6 ^a^	1.0 ± 0.6 ^b^	1.0 ± 0.6 ^b^	11.799	**<0.001**

SD: Standard deviation. ^a,b^ represent the statistically significant differences among the column groups at *p* < 0.05. Bold *p* values indicate statistical significance at the *p* < 0.05 level.

**Table 3:** Contrasting low planetary health diet quality to average–high planetary health diet quality in students by the variables.

Variables	Categories of Variables	COR (95% CI)	*p* Values for COR	AOR (95% CI)	*p* Values for AOR
Dietary energy		1.01 (1.00–1.01)	**0.007**	1.01 (1.00–1.01)	**0.001**
Genders	Females	1		1	
	Males	0.72 (0.26–2.03)	0.534	0.69 (0.24–2.04)	0.507
Age groups	<20 years	1		1	
	20–25 years	1.75 (1.20–2.55)	**0.003**	1.82 (1.07–3.12)	**0.028**
	>25 years	1.32 (0.59–2.97)	0.498	1.74 (0.72–4.25)	0.221
Income level	Low	0.96 (0.53–1.74)	0.901	1.16 (0.59–2.28)	0.665
	Adequate	1.45 (0.83–2.52)	0.191	1.25 (0.69–2.26)	0.456
	High	1		1	
Residence area	Metropolis	1		1	
	Urban	1.46 (1.00–2.13)	**0.048**	1.17 (0.74–1.87)	0.502
	Rural	0.69 (0.42–1.13)	0.141	0.63 (0.38–1.06)	0.083

AOR: Adjusted odds ratio; CI: Confidence interval; COR: Crude odds ratio; kcal: Kilocalories. Bold *p* values indicate statistical significance at the *p* < 0.05 level.

**Table 4:** Contrasting low HEI-2020 tertile to average–high HEI-2020 tertile group in students by the variables.

Variables	Categories of Variables	COR (95% CI)	*p* Values for COR	AOR (95% CI)	*p* Values for AOR
Dietary energy		1.01 (1.00–1.01)	0.518	1.00 (1.00–1.00)	0.69
Genders	Females	1		1	
	Males	0.53 (0.17–1.60)	0.258	0.50 (0.16–1.56)	0.233
Age groups	<20 years	1		1	
	20–25 years	2.13 (1.46–3.11)	**<0.001**	1.45 (0.87–2.43)	0.153
	>25 years	1.07 (0.45–2.53)	0.874	0.88 (0.35–3.22)	0.793
Income level	Low	0.59 (0.33–1.06)	0.076	0.75 (0.39–1.439	0.379
	Adequate	1.23 (0.72–2.09)	0.448	0.92 (0.52–1.62)	0.775
	High	1		1	
Residence area	Metropolis	1		1	
	Urban	2.38 (1.62–3.50)	**<0.001**	1.83 (1.15–2.91)	**0.011**
	Rural	1.18 (0.73–1.91)	0.501	1.06 (0.63–1.77)	0.840

AOR: Adjusted odds ratio; CI: Confidence interval; COR: Crude odds ratio; kcal: Kilocalories. Bold *p* values indicate statistical significance at the *p* < 0.05 level.

## Data Availability

The data that support the findings of this study are available from the corresponding author, handeyilmaz@gazi.edu.tr, upon reasonable request due to fact that it is intended to be shared only with researchers working in this field.

## Supplementary Materials

The following supporting information can be downloaded at: https://www.mdpi.com/article/10.3390/nu16091277/s1, Table S1. MANOVA test results including Healthy Eating Index-2020 and Planetary Health Diet Index total scores differences according to the sociodemographic variables interactions. Table S2. MANOVA test results including Healthy Eating Index-2020 and Planetary Health Diet Index total scores differences according to the sociodemographic variables.

## References

[1] Brown J.E. (2019). Nutrition through the Life Cycle.

[2] Vadeboncoeur C., Foster C., Townsend N. (2016). Freshman 15 in England: A longitudinal evaluation of first year university student’s weight change. BMC Obes..

[3] Mihalopoulos N.L., Auinger P., Klein J.D. (2010). The Freshman 15: Is it real?. J. Am. Coll. Health.

[4] Mortas H., Navruz-Varlı S., Bilici S. (2024). Adherence to the Planetary Health Diet and its association with diet quality in the young adult population of Türkiye: A large cross-sectional study. Nutrients.

[5] Yüksek Öğretim Kurumu (YÖK) Mezuniyet Öncesi Beslenme ve Diyetetik Eğitimi Ulusal Çekirdek Eğitim Programı-2016. https://www.yok.gov.tr/Documents/Kurumsal/egitim_ogretim_dairesi/Ulusal-cekirdek-egitimi-programlari/beslenme_ve_diyetetik.pdf.

[6] Navruz Varlı S., Bilici S. (2022). Do the sociodemographic factors and body mass index have an impact on food safety knowledge, attitudes and practices?. Tekirdağ Ziraat Fak. Derg..

[7] Helbach A., Dumm M., Moll K., Böttrich T., Leineweber C.G., Mueller W., Matthes J., Polidori M.C. (2023). Improvement of dietary habits among German medical students by attending a nationwide online lecture series on nutrition and Planetary Health (“Eat This!”). Nutrients.

[8] Bertoluci G., Masset G., Gomy C., Mottet J., Darmon N. (2016). How to build a standardized country-specific environmental food database for nutritional epidemiology studies. PLoS ONE.

[9] Schröder H., Serra-Majem L., Subirana I., Izquierdo-Pulido M., Fitó M., Elosua R. (2016). Association of increased monetary cost of dietary intake, diet quality and weight management in Spanish adults. Br. J. Nutr..

[10] Macit-Çelebi M.S., Bozkurt O., Kocaadam-Bozkurt B., Köksal E. (2023). Evaluation of sustainable and healthy eating behaviors and adherence to the planetary health diet index in Turkish adults: A cross-sectional study. Front. Nutr..

[11] Cacau L.T., Benseñor I.M., Goulart A.C., Cardoso L.O., Lotufo P.A., Moreno L.A., Marchioni D.M. (2021). Adherence to the planetary health diet index and obesity indicators in the Brazilian longitudinal study of adult health (ELSA-Brasil). Nutrients.

[12] Cacau L.T., De Carli E., de Carvalho A.M., Lotufo P.A., Moreno L.A., Bensenor I.M., Marchioni D.M. (2021). Development and validation of an index based on EAT-Lancet recommendations: The Planetary Health Diet Index. Nutrients.

[13] Marchioni D.M., Cacau L.T., De Carli E., de Carvalho A.M., Rulli M.C. (2022). Low adherence to the EAT-Lancet sustainable reference diet in the Brazilian population: Findings from the national dietary survey 2017–2018. Nutrients.

[14] Willett W., Rockström J., Loken B., Springmann M., Lang T., Vermeulen S., Garnett T., Tilman D., DeClerck F., Wood A. (2019). Food in the Anthropocene: The EAT–Lancet Commission on healthy diets from sustainable food systems. Lancet.

[15] Food and Agriculture Organization of the United Nations (2019). Dietary Guidelines and Sustainability.

[16] United Nations (2019). Department of Economic and Social Affairs, Population Division.

[17] Frank S.M., Jaacks L.M., Avery C.L., Adair L.S., Meyer K., Rose D., Taillie L.S. (2024). Dietary quality and cardiometabolic indicators in the USA: A comparison of the Planetary Health Diet Index, Healthy Eating Index-2015, and Dietary Approaches to Stop Hypertension. PLoS ONE.

[18] Krebs-Smith S.M., Pannucci T.E., Subar A.F., Kirkpatrick S.I., Lerman J.L., Tooze J.A., Wilson M.M., Reedy J. (2018). Update of the healthy eating index: HEI-2015. J. Acad. Nutr. Diet..

[19] Standen E.C., Finch L.E., Tiongco-Hofschneider L., Schopp E., Lee K.M., Parker J.E., Bamishigbin O.N., Tomiyama A.J. (2022). Healthy versus unhealthy comfort eating for psychophysiological stress recovery in low-income Black and Latinx adults. Appetite.

[20] Liu J., Micha R., Li Y., Mozaffarian D. (2021). Trends in food sources and diet quality among US children and adults, 2003–2018. JAMA Netw. Open.

[21] Liu L., Xie T., Hu Z., Liu J. (2023). Association between healthy eating index-2015 and abdominal aortic calcification: A population-based cross-sectional study. Prev. Med. Rep..

[22] Ramón-Arbués E., Granada-López J.M., Martínez-Abadía B., Echániz-Serrano E., Antón-Solanas I., Jerue B.A. (2021). Factors related to diet quality: A cross-sectional study of 1055 university students. Nutrients.

[23] WHO (2010). Fact Sheets. A Healthy Lifestyle-WHO Recommendations. https://www.who.int/europe/news-room/fact-sheets/item/a-healthy-lifestyle---who-recommendations.

[24] Merdol T. (2011). Standart Yemek Tarifeleri.

[25] Frank S.M., Jaacks L.M., Adair L.S., Avery C.L., Meyer K., Rose D., Taillie L.S. (2024). Adherence to the Planetary Health Diet Index and correlation with nutrients of public health concern: An analysis of NHANES 2003–2018. Am. J. Clin. Nutr..

[26] Semba R.D., de Pee S., Kim B., McKenzie S., Nachman K., Bloem M.W. (2020). Adoption of the ‘planetary health diet’ has different impacts on countries’ greenhouse gas emissions. Nat. Food.

[27] Scott P. (2016). Global Panel on Agriculture and Food Systems for Nutrition: Food Systems and Diets: Facing the Challenges of the 21st Century.

[28] Sautron V., Péneau S., Camilleri G.M., Muller L., Ruffieux B., Hercberg S., Méjean C. (2015). Validity of a questionnaire measuring motives for choosing foods including sustainable concerns. Appetite.

[29] Goulding T., Lindberg R., Russell C.G. (2020). The affordability of a healthy and sustainable diet: An Australian case study. Nutr. J..

[30] Pearson D., Friel S., Lawrence M. (2014). Building environmentally sustainable food systems on informed citizen choices: Evidence from Australia. Biol. Agric. Hortic..

[31] Allès B., Péneau S., Kesse-Guyot E., Baudry J., Hercberg S., Mejean C. (2017). Food choice motives including sustainability during purchasing are associated with a healthy dietary pattern in French adults. Nutr. J..

[32] Benedetti I., Laureti T., Secondi L. (2018). Choosing a healthy and sustainable diet: A three-level approach for understanding the drivers of the Italians’ dietary regime over time. Appetite.

[33] Macdiarmid J.I., Kyle J., Horgan G.W., Loe J., Fyfe C., Johnstone A., McNeill G. (2012). Sustainable diets for the future: Can we contribute to reducing greenhouse gas emissions by eating a healthy diet?. Am. J. Clin. Nutr..

[34] Reynolds C.J., Horgan G.W., Whybrow S., Macdiarmid J.I. (2019). Healthy and sustainable diets that meet greenhouse gas emission reduction targets and are affordable for different income groups in the UK. Public Health Nutr..

[35] Barosh L., Friel S., Engelhardt K., Chan L. (2014). The cost of a healthy and sustainable diet–who can afford it?. Aust. N. Z. J. Public Health.

[36] Hirvonen K., Bai Y., Headey D., Masters W.A. (2020). Affordability of the EAT–Lancet reference diet: A global analysis. Lancet Glob. Health.

[37] Cacau L.T., Hanley-Cook G.T., Huybrechts I., De Henauw S., Kersting M., Gonzalez-Gross M., Gottrand F., Ferrari M., Nova E., Castillo M.J. (2023). Relative validity of the Planetary Health Diet Index by comparison with usual nutrient intakes, plasma food consumption biomarkers, and adherence to the Mediterranean diet among European adolescents: The HELENA study. Eur. J. Nutr..

[38] Alcorta A., Porta A., Tárrega A., Alvarez M.D., Vaquero M.P. (2021). Foods for plant-based diets: Challenges and innovations. Foods.

[39] Zeng L., Ruan M., Liu J., Wilde P., Naumova E.N., Mozaffarian D., Zhang F.F. (2019). Trends in processed meat, unprocessed red meat, poultry, and fish consumption in the United States, 1999–2016. J. Acad. Nutr. Diet..

