# Supplementary material for: Sociodemographic Trends in Planetary Health Diets among Nutrition Students in Türkiye: Bridging Classroom to Kitchen
Source: Nutrients. 2024 Apr 25;16(9):1277. doi: 10.3390/nu16091277 (PMC11085215; doi:10.3390/nu16091277)
Supplement: Supplementary file 1 [file nutrients-16-01277-s001.zip › nutrients-2972438-supplementary.pdf]

**Table S1.** MANOVA test results including Healthy Eating Index-2020 and Planetary Health Diet Index total scores differences according to the sociodemographic variables interactions

| **Variables** | **Pillai's Trace** | ***F*** | ***df*** | **Error *df*** | ***p*** | **η2** |
|---|---|---|---|---|---|---|
| Age groups * Regions | 0.067 | 1.417 | 24 | 988 | 0.088 | 0.033 |
| Age groups * Residence area | 0.012 | 0.748 | 8 | 988 | 0.649 | 0.006 |
| Age groups * Income level | 0.015 | 0.918 | 8 | 988 | 0.500 | 0.007 |
| Regions * Residence area | 0.031 | 0.649 | 24 | 988 | 0.901 | 0.016 |
| Regions * Income level | 0.066 | 1.413 | 24 | 988 | 0.090 | 0.033 |
| Residence area * Income level | 0.018 | 1.100 | 8 | 988 | 0.360 | 0.009 |
| Age groups * Regions * Residence area | 0.037 | 1.177 | 16 | 988 | 0.280 | 0.019 |
| Age groups * Regions * Income level | 0.027 | 1.357 | 10 | 988 | 0.196 | 0.014 |
| Age groups * Residence area * Income level | 0.016 | 0.998 | 8 | 988 | 0.436 | 0.008 |
| Regions * Residence area * Income level | 0.076 | 1.223 | 32 | 988 | 0.185 | 0.038 |
| Age groups * Regions * Residence area * Income level | 0.010 | 0.827 | 6 | 988 | 0.549 | 0.005 |

**Table S2.** MANOVA test results including Healthy Eating Index-2020 and Planetary Health Diet Index total scores differences according to the sociodemographic variables

| Dependent Variables | df | F | η2 | *p* | Independent Variables | 95% Confidence Interval | |
|---|---|---|---|---|---|---|---|
| | | | | | | Lower Bound | Upper Bound |
| | | | | | **Age Groups** | | |
| Planetary Health Diet Index | 2 | 0.684 | 0.003 | <0.001 | <20 years[a] | 51.106 | 59.005 |
| | | | | | 20-25 years[b] | 50.751 | 56.529 |
| | | | | | >25 years[a,b] | 48.003 | 59.202 |
| Healthy Eating Index-2020 | 2 | 0.402 | 0.002 | 0.002 | <20 years[a] | 46.942 | 52.895 |
| | | | | | 20-25 years[b] | 47.320 | 51.674 |
| | | | | | >25 years[a,b] | 45.874 | 54.314 |
| | | | | | **Regions** | | |
| Planetary Health Diet Index | 6 | 1.265 | 0.015 | 0.038 | Marmara[a] | 48.672 | 57.012 |
| | | | | | Aegean[a] | 53.099 | 66.549 |
| | | | | | Mediterranean[a] | 50.798 | 61.886 |
| | | | | | Black Sea[a] | 52.202 | 65.242 |
| | | | | | Central Anatolia[b] | 44.768 | 53.199 |
| | | | | | Eastern Anatolia[a,b] | 44.059 | 59.367 |
| | | | | | Southeast Anatolia[a,b] | 45.511 | 59.730 |
| Healthy Eating Index-2020 | 6 | 1.048 | 0.013 | 0.007 | Marmara[a] | 44.917 | 51.201 |
| | | | | | Aegean[a,b] | 47.210 | 57.345 |
| | | | | | Mediterranean[a,b] | 46.265 | 54.621 |
| | | | | | Black Sea[a,b] | 43.181 | 53.008 |
| | | | | | Central Anatolia[b] | 44.314 | 50.668 |
| | | | | | Eastern Anatolia[a,b] | 43.598 | 55.135 |
| | | | | | Southeast Anatolia[a,b] | 49.199 | 59.914 |
| | | | | | **Residence Area** | | |
| Planetary Health Diet Index | 2 | 0.696 | 0.003 | <0.001 | Metropolis[a] | 47.811 | 55.824 |
| | | | | | Urban[b] | 50.402 | 58.325 |
| | | | | | Rural[a] | 52.137 | 58.959 |
| Healthy Eating Index-2020 | 2 | 1.076 | 0.004 | <0.001 | Metropolis[a] | 47.269 | 53.308 |
| | | | | | Urban[b] | 44.601 | 50.572 |
| | | | | | Rural[a] | 48.528 | 53.669 |
| | | | | | **Income Level** | | |
| Planetary Health Diet Index | 2 | 1.701 | 0.007 | 0.006 | Low[a] | 52.721 | 61.594 |
| | | | | | Adequate[b] | 49.480 | 55.431 |
| | | | | | High[a] | 49.066 | 57.389 |
| Healthy Eating Index-2020 | 2 | 0.724 | 0.003 | 0.035 | Low[a] | 47.242 | 53.929 |
| | | | | | Adequate[b] | 47.498 | 51.982 |
| | | | | | High[a,b] | 45.810 | 52.083 |

[a, b]represent the statistically significant differences among the column groups at $p < 0.05$.
